# B cell class switching in intestinal immunity in health and disease

**DOI:** 10.1111/sji.13139

**Published:** 2022-01-12

**Authors:** Aaron Fleming, Tomas Castro‐Dopico, Menna R. Clatworthy

**Affiliations:** ^1^ Molecular Immunity Unit Department of Medicine Cambridge Institute of Therapeutic Immunology and Infectious Diseases University of Cambridge Cambridge UK; ^2^ The Francis Crick Institute London UK; ^3^ Cellular Genetics Wellcome Trust Sanger Institute Hinxton UK; ^4^ NIHR Cambridge Biomedical Research Centre Cambridge UK

**Keywords:** antibodies, B cells, intestinal immunity

## Abstract

The gastrointestinal tract is colonized by trillions of commensal microorganisms that collectively form the microbiome and make essential contributions to organism homeostasis. The intestinal immune system must tolerate these beneficial commensals, whilst preventing pathogenic organisms from systemic spread. Humoral immunity plays a key role in this process, with large quantities of immunoglobulin (Ig)A secreted into the lumen on a daily basis, regulating the microbiome and preventing bacteria from encroaching on the epithelium. However, there is an increasing appreciation of the role of IgG antibodies in intestinal immunity, including beneficial effects in neonatal immune development, pathogen and tumour resistance, but also of pathological effects in driving chronic inflammation in inflammatory bowel disease (IBD). These antibody isotypes differ in effector function, with IgG exhibiting more proinflammatory capabilities compared with IgA. Therefore, the process that leads to the generation of different antibody isotypes, class‐switch recombination (CSR), requires careful regulation and is orchestrated by the immunological cues generated by the prevalent local challenge. In general, an initiating signal such as CD40 ligation on B cells leads to the induction of activation‐induced cytidine deaminase (AID), but a second cytokine‐mediated signal determines which Ig heavy chain is expressed. Whilst the cytokines driving intestinal IgA responses are well‐studied, there is less clarity on how IgG responses are generated in the intestine, and how these cues might become dysfunctional in IBD. Here, we review the key mechanisms regulating class switching to IgA vs IgG in the intestine, processes that could be therapeutically manipulated in infection and IBD.

## 1 INTRODUCTION

Humoral immunity plays a critical role in the gastrointestinal tract, an organ colonized by trillions of commensal microorganisms, collectively known as the microbiome. The microbiome contributes to physiological processes, including nutrient absorption and barrier protection, and occupies a tolerogenic nutrient‐rich niche that enables a symbiotic relationship with the host.^1^, ^2^ Given the large density of microorganisms, particularly in the lower gastrointestinal tract, the intestinal immune system must strike a balance, tolerating commensals whilst preventing the invasion of pathogens. Disruption of this balance leads to dysbiosis and inflammation, and is associated with a variety of intestinal and systemic disorders.^3^ In this context, immunoglobulins (Ig) are produced in the gut mucosa and secreted into the gut lumen, mediating tolerance to commensals and shaping the composition of the microbiome through a non‐inflammatory process primarily known as immune exclusion.^4^, ^5^ IgA is the dominant antibody isotype at mucosal surfaces, with large quantities (3–5 g per day in humans)^6^ secreted into the gut lumen. In addition to IgA, other antibody isotypes also contribute to intestinal humoral immunity. IgM is transcytosed and secreted into the gut lumen in humans with similar efficiency to IgA, and it participates in immune exclusion.^7^ Furthermore, despite the limited presence of IgG^+^ plasma cells in the healthy gut, elevated IgG‐expressing cells have long been noted in the mucosa of patients with intestinal inflammation.^8^, ^9^, ^10^, ^11^ Recently, there has been renewed interest in the role of IgG in intestinal host‐microbe interactions both in health and disease.^4^, ^12^ However, the signals that regulate class switching of naïve B cells to IgG‐producing plasma cells and memory B cells in the intestine are not fully understood. Here, we consider the differing effector functions of IgA vs IgG, review the molecular processes underpinning class‐switch recombination (CSR) and discuss the factors that determine class switching to IgA vs IgG in the intestine in health and disease.

## 2 B CELL SUBSETS AND ACTIVATION

B cell antibody production is shaped by the nature of the stimulating antigen and the environmental context in which that antigen is encountered. Protein antigens induce high‐affinity antibody responses within secondary lymphoid organs (SLOs), such as lymph nodes, spleen and gut‐associated lymphoid tissue (GALT), and require cognate interactions with antigen‐specific CD4 T cells, a process termed as T cell–dependent (TD) antibody production. Following T cell interaction, a subset of activated B cells generate short‐lived extrafollicular plasmablasts. Others form germinal centres (GCs), where they undergo class switching from IgM to IgG, IgA or IgE expression and affinity maturation, culminating in the emergence of long‐lived plasma cells and memory B cells. T cell–independent (TI) responses are mounted against carbohydrate/polysaccharide antigens in areas largely, but not completely, devoid of germinal centres.^13^ These microscopically visible solitary isolated lymphoid tissues (SILTs) include cryptopatches as well as immature and mature isolated lymphoid follicles (ILFs).^14^, ^15^ Here, activated B cells and plasma cells exhibit lower levels of somatic hypermutation (SHM), indicative of GC independence. TI B cell responses can be broadly divided into two classes based on the antigen in question: TI type 1 responses are induced by antigens that polyclonally activate B cells, such as potent TLR or coreceptor signalling, and TI type 2 responses mediated by B cell receptor (BCR) recognition of multivalent epitopes.^16^ In addition to BCR activation, TI type 2 responses require accessory signals to promote the development of antigen‐specific plasma cells. TI responses by marginal zone B cells in the spleen can also be induced in both a contact‐dependent and contact‐independent manner by a broad range of innate cells, including neutrophils,^17^ dendritic cells (DCs)^18^ and mast cells.^19^ These innate ‘B helper cells’ have been shown to be important (in mice) for protection from a range of pathogens including *S typhimurium* ^20^ and West Nile Virus.^21^ The reliance on innate cells in these responses has led to the proposal that they may constitute a “TI‐3” response distinct from TI type 1 and TI type 2 responses.^22^

Further complexity arises when considering B cells themselves. In mice, the B cell lineage is broadly divided into two cell types–B1 and B2 cells–annotated according to their ontology, with B1 cells evident in the early prenatal period prior to the development of bone marrow, from which B2 cells arise. Post‐natally, B2 cells form the major B cell population, giving rise to follicular B cells that reside within SLOs that participate in both TD and TI responses, as well as marginal zone (MZ) B cells–a specialized splenic B cell subset that produces TI antibody in response to blood‐borne encapsulated bacteria.

Innate‐like B1 cells, along with MZ B cells, mainly express germ line–encoded antigen receptors with limited diversity and also participate in TI responses.^23^ In mice, they are further subdivided into B1a and B1b cells, based on the presence or absence of CD5 expression, respectively, and are principally located in the peritoneal and pleural cavities, and to a lesser extent, in lymphoid tissues.^24^ B1a cells produce low‐affinity polyreactive natural antibodies, mainly IgM and IgG3 in mice, with reactivity against self and foreign carbohydrate antigens, whilst B1b cells contribute to adaptive antibody responses to TI antigens, for example, in response to infection.^25^ Their presence and phenotype in humans are debated,^26^ with a CD20^+^ CD27^+^ CD43^+^ CD70^−^ population initially identified as the human counterpart to murine B1 cells subsequently contested as representing preplasmablasts.^27^, ^28^, ^29^ However, CD5 is not a specific marker for B1 cells in humans, and their existence in humans is still debated.

## 3 ANTIBODY EFFECTOR FUNCTION–IgA VS IgG

The major output of B cell activation is an antibody‐secreting cell. Each antibody isotype exhibits distinct effector functions; these include variable domain‐dependent functions such as bacterial/viral/toxin neutralization and Fc domain‐dependent functions, including complement activation, Fc receptor engagement and epithelial cell transcytosis. Therefore, the generation of specific Ig isotypes is tightly regulated to enable a humoral response optimized for the current, prevalent immune challenge. In considering why IgA dominates at mucosal surfaces, and why IgG, although the major circulating antibody, may be expressed in the gut in health and disease, it is instructive to consider the differing effector functions of IgA vs subclasses of IgG.

### 3.1 IgA antibody structure and effector function

**FIGURE 1:**
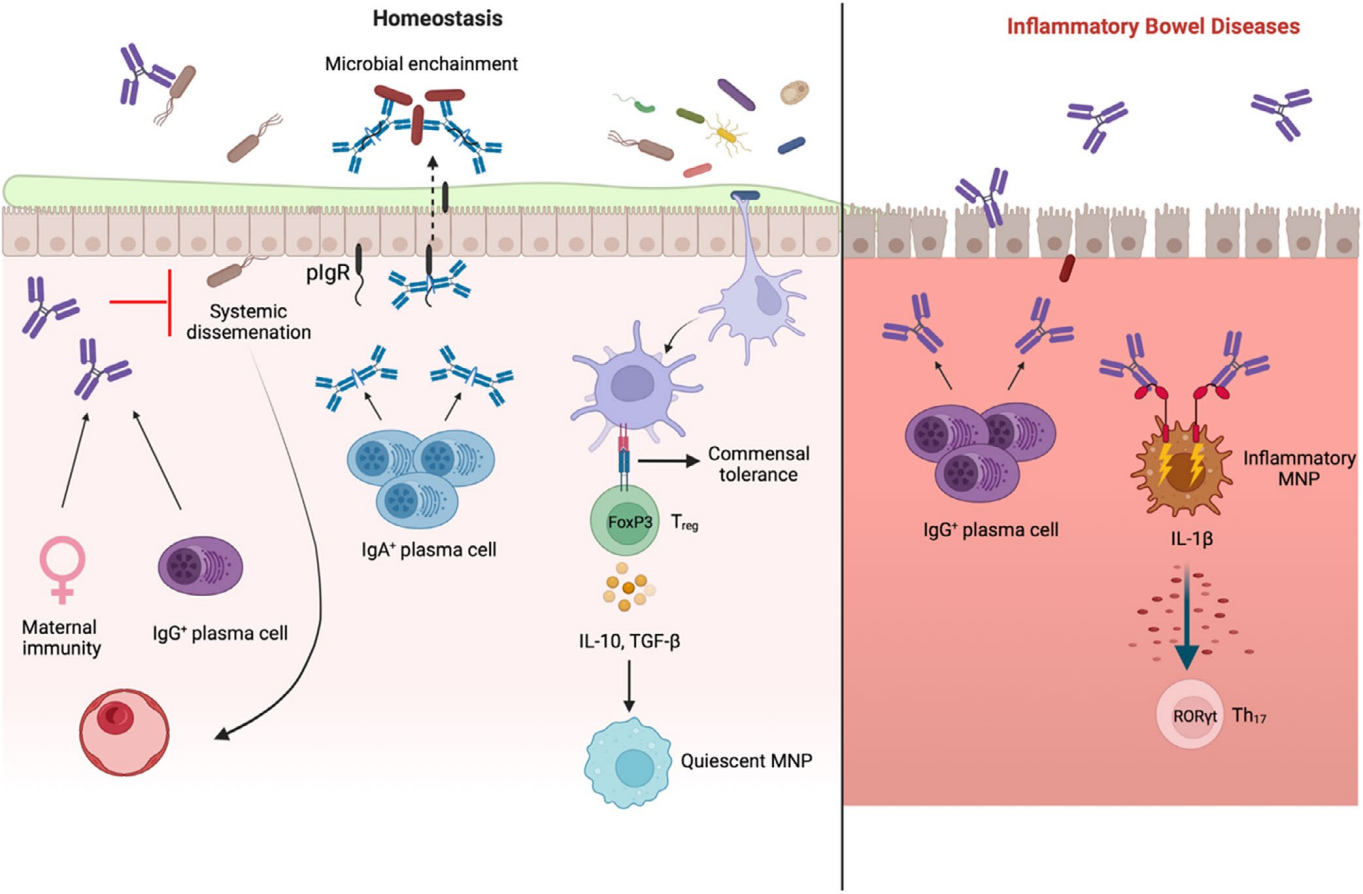
Intestinal IgA and IgG in health and disease: During homeostasis (left), sIgA is the predominant antibody present in the gut mucosa. IgA^+^ plasma cells, quiescent MNPs and FoxP3‐expressing regulatory T cells form an immunotolerant triad in the tract, which directs IgA responses mostly against commensal microbes. sIgA is transported from the basolateral side of epithelial cells by the polymeric immunoglobulin receptor (pIgR), which attaches a secretory component to the IgA, releasing it into the intestinal lumen as sIgA. Here, sIgA mediates homeostasis by preventing commensal activation of epithelial cells and warding off potential pathobionts, including by enchaining bacteria. There is a relative paucity of anti‐commensal IgG during homeostasis, some of which is maternallyderived, and functions to prevent systemic spread of potential pathogens. In conditions such as UC and CD (right), IgG‐secreting B cells are induced and appear in large numbers. When gross epithelial barrier breach occurs, IgG can bind both pathogenic and commensal bacteria, causing crosslinking and activation of resident MNPs via FcγR interactions, induction of proinflammatory IL‐1β, and engagement of RORγt‐expressing Th_17_ cells

Mice possess a single IgA subclass, whilst humans and other great apes have two subclasses, IgA1 and IgA2. IgA1 dominates in human serum, but in the intestine, the relative abundance of IgA1 vs IgA2 varies along the length of the digestive tract, with the IgA1:IgA2 ratio decreasing from 3:1 in the proximal small intestine to 1:3 in the colon.^4^, ^30^ IgA2 possess a shorter hinge region than IgA1, which makes it less susceptible to *Streptococcus*‐derived proteases,^31^ and this may increase its durability in the microbe‐rich colon.^32^, ^33^ Polymeric IgA, consisting of predominantly dimeric IgA covalently connected by the joining or J chain, binds to the polymeric Ig receptor (pIgR) on the basolateral side of intestinal epithelial cells, before being internalized and transcytosed to the apical surface.^4^ Here, it is proteolytically cleaved and released as secretory IgA (sIgA) into the gut lumen^6^ (Figure 1). Class‐switched IgA^+^ B cells are mainly generated in the gut‐associated lymphoid tissue (GALT), including Peyer's patches and mesenteric lymph nodes, with long‐lived IgA^+^ plasma cells seeding from there to the intestinal lamina propria.

IgA mainly mediates its effector functions by binding to microbes and inhibiting epithelial invasion, as well as trapping or enchaining bacteria within the mucus layer, thereby promoting tolerance of intestinal commensals^34^ (Table 1). Fc‐mediated effector functions (particularly in the gut) are limited; IgA weakly activates complement, it does not mediate NK cell–mediated cytotoxicity, and overall, it has weak opsonising capability. In humans, there is a specific IgA Fc receptor (FcαRI (CD89)), which is expressed on myeloid cells, particularly neutrophils, but also on monocytes and some macrophage subsets.^35^ However, mice do not express FcαRI, but other IgA‐binding receptors include the transferrin receptor (CD71), asialoglycoprotein (ASGP)‐R, FcαμR and the polymeric IgR.^35^ Of note, the induction of systemic protective IgA antibodies has been described following vaccination and in a number of bacterial and viral infections.^36^, ^37^ In this context, IgA does have some capacity to opsonize viruses or bacteria for uptake by phagocytes and may induce TRIM21‐dependent proteasomal destruction of intracellular virus.^36^, ^37^, ^38^, ^39^

**TABLE 1:** Antibody effector functions (human)

	Effector functions	IgG1	IgG2	IgG3	IgG4	IgA
Fc dependent	Complement activation	++	+	+++	−	+
	Opsonization	+++	+/−	++	−	+
	ADCC	++	−	++	−	−
V‐region–dependent	Neutralization	++	++	++	++	++

Note

ADCC = NK cell–mediated antibody‐dependent cellular cytotoxicity.

### 3.2 IgG antibody structure and effector function

There are four IgG subclasses in humans (IgG1‐4) and mice (IgG1, IgG2a/c, IgG2b and IgG3) (Table 1). Human IgG1 is the most abundant and predominantly targets soluble protein antigens and membrane proteins.^40^ The generation of IgG1 is largely TD, and it exhibits potent effector functions, including complement activation and antibody‐mediated cellular cytotoxicity.^41^ In mice, the effector profile of IgG2a and IgG2b is most similar to human IgG1 and also show strong effector function in vivo.^42^ Human IgG2 responses (IgG3 in mice) are almost completely restricted to TI bacterial capsular carbohydrates, although anticarbohydrate IgG antibodies of other subclasses do exist.^43^ IgG2 and IgG4 antibodies have a short, rigid hinge region compared with IgG1 and 3, resulting in impaired antibody flexibility, and this influences affinity for IgG Fc receptors (FcγRs) and C1q. Human IgG3 antibodies are the most effective subclass in terms of their activating effector functions, with avid complement‐activating capacity and affinity for activating FcγRs, but they exhibit a lower half‐life than other IgG subtypes due to impaired recycling via the neonatal Fc receptor (FcRn).^44^ Finally, IgG4 is associated with induction by long‐term exposure to antigens in a non‐infectious setting, as observed in immune responses to allergens or parasitic infection.^40^ IgG4 has relatively high affinity for the inhibitory receptor FcγRIIB, does not fix complement, exhibits an ability to spontaneously dissociate and form bispecific antibodies^45^ and has the capacity to compete with IgE for allergens. It is therefore proposed to act as an inhibitor of effector responses.^46^ Beyond subclass, post‐translational modification of the Fc region of IgG, most notably via *N*‐linked glycosylation, fine tunes FcγR affinity and complement activity.^47^, ^48^, ^49^, ^50^ Each IgG heavy chain carries a single covalently attached biantennary *N*‐glycan at the asparagine 297 residue of the Fc fragment Cγ2 domains, with over 900 IgG glycoforms possible.^51^ Biantennary complexes can contain additional bisecting *N*‐acetylglucosamine (GlcNAc), core fucose, galactose and sialic acid residues.^52^

**FIGURE 2:**
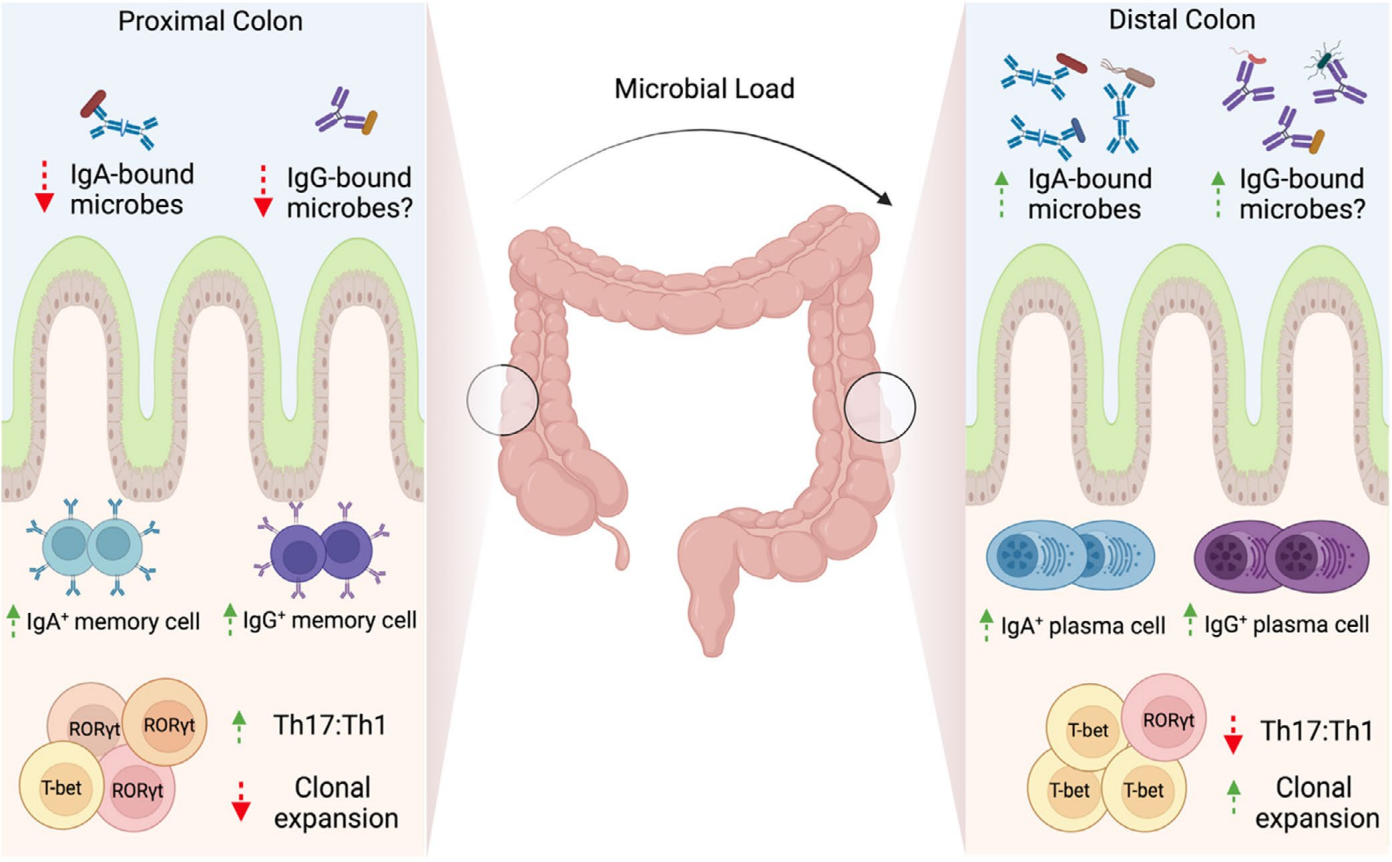
IgG and IgA cell profiles in the colon: The microbial load of the colon is known to increase from proximal to distal in both mice and man. Memory B cells predominate in the proximal colon, with more plasma cells of both the IgA and IgG variety in the distal colon. This plasma cell–rich profile of the distal colon is accompanied by a decreased Th17:Th1 ratio and increased clonal expansion of CD4^+^ T cells vs the proximal colon. More IgA‐bound microbes can also be found in the lumen of the distal colon, with potentially more IgG‐bound microbes to be found here also

Although IgA dominates in the intestine, commensal‐reactive IgG2b and IgG3 have been identified in GALT in healthy mice.^53^ In homeostatic, non‐inflamed human colon, scRNA sequencing has shown that CD38‐expressing IgG^+^ plasma cells are enriched in the distal sigmoid colon compared with the more proximal caecum and transverse colon^33^ (Figure 2). This spatial segregation of IgG plasma cells in the colon was coincident with an increased bacterial diversity, Th1:Th17 ratio, IgA plasma cells and CD4^+^ T cell clonal expansion in the distal colon. IgG plasma cells were predominantly IgG1 and IgG2; however, memory B cells predominately expressed IgG1 but little IgG2, indicating that IgG isotype expression is distinct between plasma or memory B cell fates. Although this study did not address IgG binding to commensal bacteria, distal colon‐resident commensal microbes were more likely to be bound by IgA than proximal colon‐resident microbes, suggesting that spatial differences in plasma cell abundances are functionally relevant and likely to have specific effects on the local predominance of antibody‐bound commensal microbes.^33^

Overall, relative to IgA, IgG is a far more proinflammatory immunoglobulin. As noted above, most IgG subclasses can activate complement, but many immune activating effects of IgG are mediated by binding to FcγRs. In humans, there are several activating receptors (FcγRIIA, FcγRIIC, FcγRIIIA and FcγRIII/) and a single inhibitory receptor, FcγRIIB, which plays a critical role in suppressing IgG‐mediated inflammation.^54^, ^55^ FcγRs are expressed on almost every immune cell type, including neutrophils, monocytes, macrophages, DCs, mast cells, natural killer (NK) cells and B cells, and this pattern of expression enables IgG to engage almost every facet of the immune system, underpinning its potent proinflammatory potential. Most FcγRs are low‐to‐medium affinity for IgG, requiring cross‐linking of several receptors into signalling synapses on the cell surface in order to initiate productive signalling. This is achieved through the formation of immune complexes (IC) between antigen and antigen‐specific IgG or by IgG‐opsonised cells. The inhibitory receptor, FcγRIIB, acts as an additional regulatory mechanism to suppress IgG‐mediated inflammation, although its expression is heterogeneous across cells of the immune system and subject to regulation by various stimuli, particularly by the cytokine milieu.^56^, ^57^ The ratio of activating to inhibitory FcγRs on any given cell is known as the activating/inhibitory (A/I) ratio, and its context‐specific modulation allows for appropriate immune responses to be raised.^54^, ^58^ Genetic polymorphisms in human *FCGR* genes that alter receptor expression or function are frequently associated with differential susceptibility to both infection and autoimmunity, including inflammatory bowel disease.^55^, ^58^, ^59^ Differences in IgG glycosylation can also alter affinity for activating vs inhibitory FcγRs^60^, ^61^, ^62^, ^63^; for example, defucosylation increases the binding affinity of IgG for activating FcγRIIIA (but not FcγRIIB) 10–50 fold.^64^

IgG functions in the intestine in homeostasis include protection against infectious challenge^65^, ^66^, ^67^, ^68^ and allergic intolerance,^69^ neonatal immune development^53^, ^70^ and tumour resistance.^71^ Conversely, chronic inflammation of the intestine in inflammatory bowel disease (IBD), a clinically heterogenous group of disorders, may be driven by the proinflammatory effects of IgG, activating local FcγR‐expressing cells^72^, ^73^, ^74^, ^75^ (Figure 1).

## 4 CLASS‐SWITCH RECOMBINATION

The ability to change the antibody isotype produced, in‐line with the nature and context of the immunological challenge, is a key feature of humoral immunity. The process of selecting the heavy chain which will confer the most appropriate effector function profile for the current challenge is central to achieve an effective response.

### 4.1 Molecular processes underpinning CSR

**FIGURE 3:**
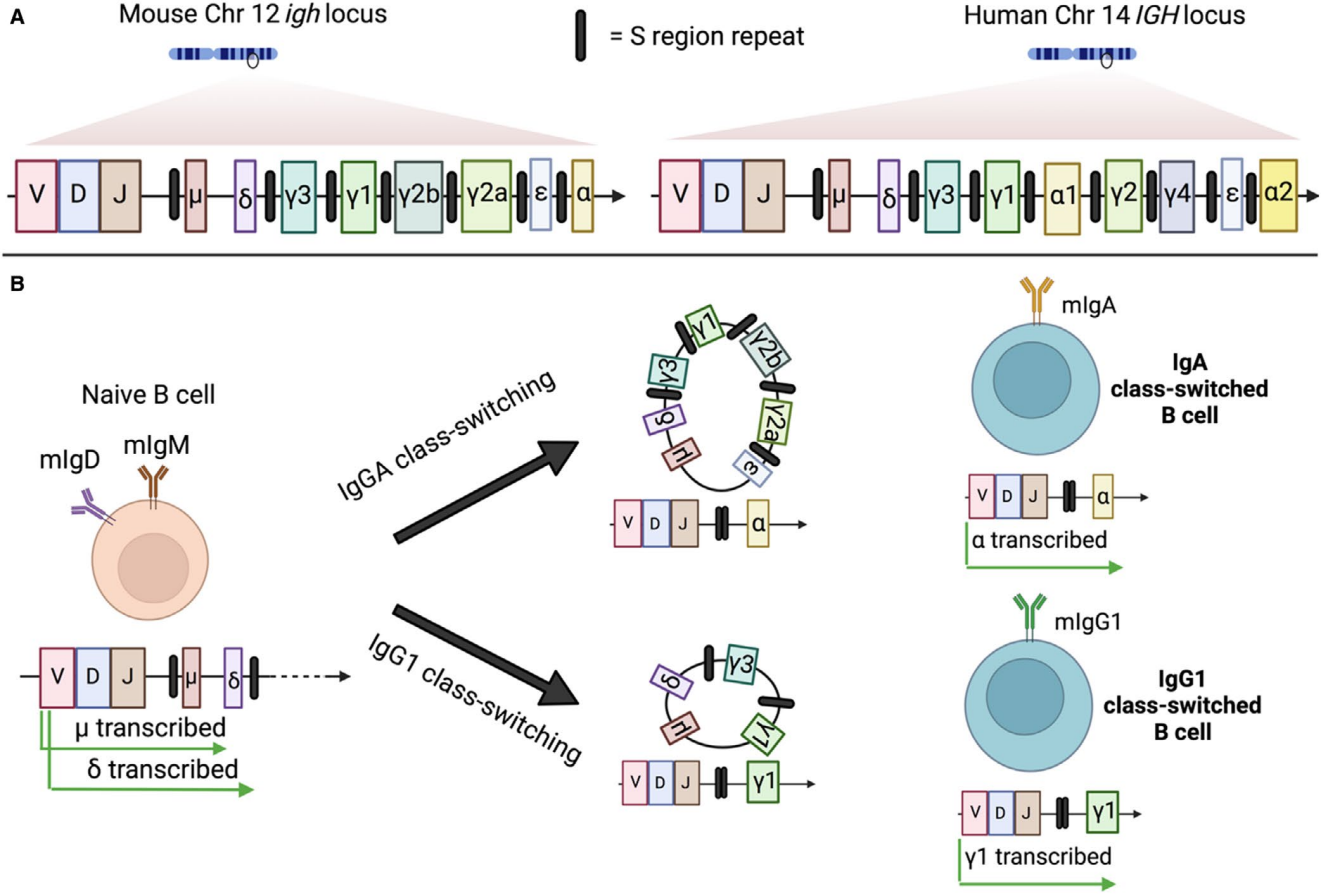
Class‐switch recombination: (A) The locus for the heavy chain constant region for both mouse and human DNA is depicted. The genes encoding the heavy chain constant regions are arrayed alongside each other downstream of the VDJ segments, which defines the antigen specificity of the BCR. All the heavy chain constant regions, with the exception of the δ gene, are flanked at their 5′ end by switch (S) regions, which are responsible for governing the AID‐mediated recombination events required to switch isotype class during activation. (B) Naïve B cells that have not encountered their cognate antigen (left) express both membrane‐bound IgM and IgD. Upon antigen encounter and T cell help, class switching begins, substituting these existing transcribed constant domains of the BCR for other isotypes. Mouse B cell isotype switching to IgA or IgG1 is presented as an example of class‐switch recombination. Depending on the cytokine and T cell signals delivered to the B cell during activation, it will preferentially excise intervening constant region genes by a recombination event mediated by S region joining (middle), resulting in the placement immediately downstream of the VDJ segment of the desired isotype (right)

CSR occurs by an intrachromosomal deletion recombination event with the replacement of the default expressed C_μ_ exon cluster in naïve B cells with Cγ, Cϵ or Cα, for IgG, IgE and IgA, respectively (Figure 3).^76^ The exact molecular mechanisms governing the intrachromosomal DNA recombination events have been reviewed extensively elsewhere.^77^, ^78^, ^79^ Briefly, the exons for the different constant heavy chains that specify isotype class are located downstream of the heavy chain VDJ sequences. All of the constant genes, with the exception of δ, are preceded by switch (S) regions, which vary in length depending on the constant gene in question, but are characterized by the presence of highly repetitive nucleotide sequences.^80^, ^81^ CSR begins when intronic promoters' upstream of the targeted S region begin transcribing germ‐line transcripts (GLTs), untranscribed mRNA units consisting of the S region promoter, S region and constant region gene. The unique repetitive nucleotide sequences in the S region lead to the formation of bubble‐like structures called R loops in the DNA as transcription proceeds, whereby the non‐template DNA strand is displaced, forming single‐stranded DNA, which is the substrate of activation‐induced cytidine deaminase (AID).^82^, ^83^ Accessory proteins then allow for the formation of double‐stranded breaks through either base excision repair or mismatch repair depending on the proximity of the AID‐induced single‐stranded breaks to one another.^84^ The donor region, which is invariably the Sμ region, is then joined to the acceptor S region of the constant gene being switched in, and the intervening DNA sequences of other constant genes are excised (Figure 3). The somatic editing nature of class‐switch recombination makes it irreversible.

### 4.2 Signals determining CSR

A variety of immune‐ and pathogen‐derived signals regulate the initiation and isotype selection of CSR, playing a critical role in determining the dominant antibody isotype produced in different contexts. Broadly, B cell–intrinsic CD40 and/or Toll‐like receptor signalling act as critical primary signals in CSR involved in the induction of the enzyme AID and other CSR factors,^79^ whilst T cell‐ and DC‐derived cytokines provide secondary signals that determine the fate of CSR, namely the isotype selected.

#### 4.2.1 Primary signals

CD40 ligand (CD40L) and/or TLR stimulation provide essential signals to initiate B cell proliferation and CSR.^79^, ^85^ In TD antibody responses, antigen‐activated B cells migrate to the T‐B border in secondary lymphoid organs where they form cognate interactions with DC‐primed T cells destined to become T follicular helper (Tfh) cells,^86^ as well as CD40L expression; these Tfh cells are essential providers of B cell–activating cytokines, particularly IL‐4 and IL‐21, that direct GC activity, CSR and the emergence of high‐affinity plasma cells and memory B cells.^87^, ^88^ Recently, Tfh cells were found to progressively secrete IL‐21 and IL‐4 for the induction of high‐affinity BCR clones and the development of Blimp‐1–dependent plasma cells, respectively.^89^ Moreover, additional stimuli that induce B cell proliferation can be delivered from antigen‐capturing stromal follicular dendritic cells (FDCs), including CXCL13,^90^ B cell–activating factor (BAFF)^90^ and cholesterol metabolites.^91^

In TI antibody responses, PAMPs acting through TLRs can induce AID expression in an NFkB‐dependent manner, with TLR signalling involved in polysaccharide‐specific IgG generation and immune responses to encapsulated bacteria. TLR1‐2, TLR4, TLR7 and TLR8 can synergise with the BCR to induce class switching to IgG3 and other isotypes in the absence of T cell help, with murine IgG3 levels relatively unaffected by the absence of CD40 or T cells.^23^, ^92^, ^93^ Lipopolysaccharide (LPS), found in the outer membrane of Gram‐negative bacteria, is the only known microbial product that can directly induce CSR through simultaneous TLR4 ligation and BCR cross‐linking in murine B cells, although not in human B cells, and can potently activate B1 and MZ B cells.^94^

In the absence of T cells, B cells participating in TI type 2 responses may receive additional proactivation and survival signals that promote B cell activation and proliferation, such as BAFF and a proliferation‐inducing ligand (APRIL).^16^, ^17^ These tumour necrosis factor (TNF) ligand superfamily members engage the BAFF receptor (BAFF‐R), B cell maturation antigen (BCMA) and transmembrane activator and CAML interactor (TACI) to stimulate AID expression.^92^, ^95^ BAFF and APRIL are released in response to TLR stimulation by several cell subsets, including mononuclear phagocytes, neutrophils, eosinophils, ILCs, DCs, FDCs and epithelial cells.^17^, ^90^, ^96^, ^97^, ^98^, ^99^, ^100^

#### 4.2.2 Secondary signals

Naïve B cells can switch to any isotype, an event that is controlled by immune cells, such as T cells and DCs, through the secretion of specific cytokines. These cytokines dictate the isotype most appropriate for eliciting pathogen/antigen‐tailored responses in the context of type 1, 2 or 17 immunity. In mice, Th2‐associated IL‐4 supports class switching to IgG1 and IgE in the presence of CD40L, whilst TGF‐β plays a crucial role in the induction of IgA. TGF‐β can induce histone modification at the Sα region that makes it more amenable to the CSR machinery.^101^ In addition to TGFβ, BAFF, APRIL, IL‐10 and IL‐6 have also been documented to support IgA CSR.^87^, ^102^ In contrast, Th1‐associated IFNγ induces class switching to IgG2a in vitro and in vivo.^103^, ^104^, ^105^ More recently, Th17 cells have been demonstrated to support IgG CSR in vivo: IL‐17A and IL‐21 promote IgG2a/IgG3 and IgG1/2b CSR, respectively, in mice.^106^

Beyond class‐switching itself, T cell–derived cytokines can also influence IgG glycosylation patterns at the asparagine 297 residue on each IgG heavy chain. A recent elegant study demonstrated that in response to IL‐23, Th17‐derived IL‐22 and IL‐21 could regulate IgG sialylation and augment IgG inflammatory activity in a murine model of rheumatoid arthritis (RA).^107^ Therefore, cytokines play a crucial role in determining both Ig isotype CSR and the inflammatory potential of IgG. The relevance of these pathways to intestinal IgA and IgG class switching will be discussed below.

## 5 INTESTINAL CLASS SWITCHING IN HEALTH

**FIGURE 4:**
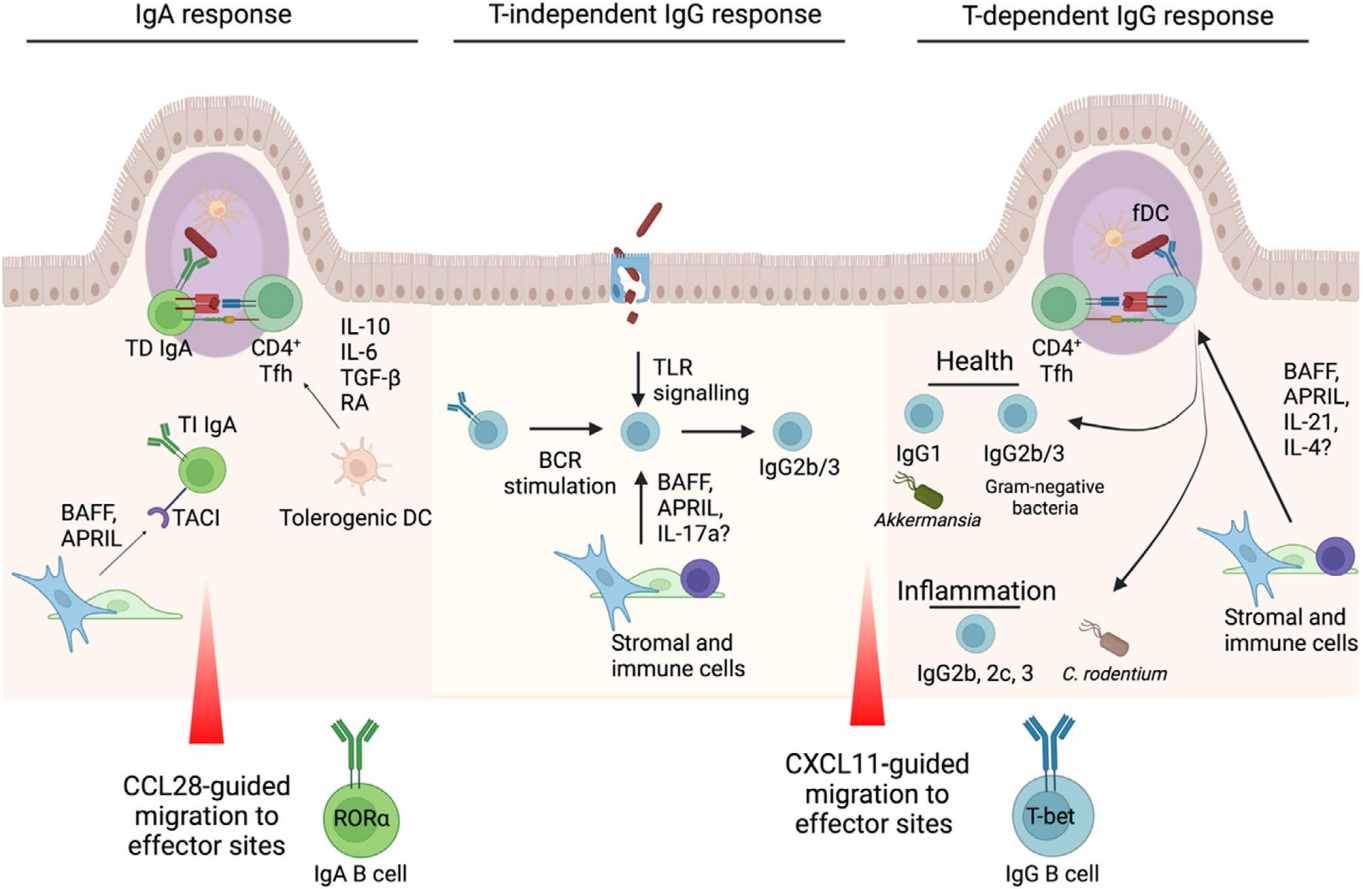
IgA vs. IgG generation and maintenance in the intestinal tract: IgA responses, which can be both T cell–dependent and –independent. In T‐dependent responses in the Payer's patches, a variety of cytokines including IL‐10, IL‐6, TGF‐β and retinoic acid (RA) from DCs drive IgA induction via CD4^+^ Tfh cells. BAFF and APRIL can mediate T‐independent responses outside the Peyer's patches via TACI binding on B cells. Once generated, IgA^+^ B cells can migrate from their inductive sites to effector sites via chemokines including epithelial‐derived CCL28, where they express the transcription factor RORα. In mice, intestinal IgG2b/3 CSR in health (middle) has been shown to be largely independent of T cells and dependent on B cell–intrinsic TLR signalling. BAFF and APRIL, released by haematopoietic and stromal cells following microbial stimulation, can promote TI AID expression in B cells, while IL‐17A can support IgG3 CSR. The species of microbes targeted by mucosal IgG responses differ between experimental conditions, suggesting a plastic response highly dependent on the composition of the microbiota. In healthy mice, T cell–dependent IgG responses towards the microbiota that have been identified include IgG1 targeting of *Akkermansia muciniphila*, as well as cross‐reactive IgG2b/3 responses towards Gram‐negative bacteria that arise following dissemination of intestinal bacteria and provide protection against systemic infection. Inflammatory T cell–dependent IgG responses are also critical for elimination of *C rodentium* in mice, with similar TD mechanisms likely to be involved in inflammatory bowel disease in humans and mice

IgA class switching in the gut requires the coordinated interaction of intestinal epithelium, DCs, macrophages and regulatory T cells, which enables commensal microbes and antigens to be sampled and to subsequently induce B cell class switching in the context of a homeostatic milieu rich in IL‐10 and TGFβ^4^, ^6^, ^87^, ^108^ (Figure 4). The signals regulating IgA class switching in the gut have been extensively studied and reviewed.^4^, ^6^, ^108^, ^109^, ^110^ Here, we discuss the major principles of intestinal IgA CSR and how these observations may be relevant to understanding the regulation of intestinal IgG response in health and disease.

### 5.1 Microbial antigens and T cell help in IgA vs IgG responses

GALT, in particular Peyer's patches of the small intestine, are the major site of IgA+B cell generation in the gut.^4^, ^6^, ^108^ TD responses arising from B2 cells are directed towards protein antigens, occur within Peyer's patches and mesenteric lymph nodes (MLNs) and rely on CD40L‐CD40 interactions with Tfh cells. In contrast, TI IgA responses occur within the GALT and MLN, as well as in non‐lymphoid tissues and can arise from both B1b and B2 cells in the presence of BAFF and APRIL.^108^, ^111^, ^112^, ^113^ Many enteric pathogens elicit TD high‐affinity IgA responses, whilst commensals can elicit both TD and TI responses.

Peyer's patches are composed of several B cell follicles on a network of follicular dendritic cells, with interfollicular regions of DCs and T cells directly underlying the follicle‐associated epithelium and subepithelial dome.^114^ Germinal centres are constitutively present in Peyer's patches,^6^ resulting from continuous exposure to microbial antigens that are trafficked through M cells. GC formation in Peyer's patches and the MLN is strictly dependent on T cells, with Tfh cells potentially arising from other CD4^+^ T cell subsets due to their plasticity.^115^, ^116^, ^117^, ^118^ However, non‐cognate T‐B interactions have been shown to be sufficient for GC formation and SHM in Peyer's patches. B cells expressing surrogate Ig receptors can become activated to form GCs in Peyer's patches, in the presence of non‐cognate T cell help and microbiota‐derived signals.^119^ Therefore, under certain circumstances, BCR‐independent B cell activation can support T cell–dependent IgA production in Peyer's patches.

GALT GCs and IgA^+^ plasma cell formation are severely impaired in CD40‐ and T cell–deficient mice.^115^, ^120^, ^121^, ^122^ Strikingly, however, total levels of IgA‐opsonised commensal microbes remain largely unaffected in these mice, although they exhibit impaired antigen‐specific IgA responses to protein antigens, such as cholera toxin.^121^, ^122^, ^123^ Whilst the microbial species targeted in T cell–deficient mice differs significantly from WT mice, including an inability to target segmented filamentous bacteria (SFB), this was found to be independent of CSR or SHM. In a series of elegant experiments, commensal reactive IgA was found to derive from T cell–independent B1b and T cell–responsive B2 cells, with both responses covering overlapping and diverse bacterial taxa,^115^ although the majority of IgA^+^ plasma cells showed signs of SHM. Therefore, whilst T‐B interactions are not required for low‐affinity TI IgA responses, they are required to mount specific IgA responses to microbes and orally administered TD antigens.

Although dominated by IgA, intestinal B cell IgG class switching occurs at a low level during homeostasis. Koch et al demonstrated commensal‐reactive IgG2b and IgG3 responses within MLN and Peyer's patches in mice.^53^ These antibodies were largely generated independently of T cells but required B cell–intrinsic TLR signalling. This is consistent with the known requirements for TI IgG3 responses in mice, which predominantly target carbohydrate antigens.^93^ Furthermore, IgG2b/IgG3^+^ B cells exhibited cell‐surface expression phenotype consistent with plasma cells and B1 cells, including CD138 and CD43, respectively. In a complementary study, gut microbiota‐induced IgG antibodies (predominantly IgG2b and IgG3) were shown to mediate systemic protection against *E coli* and *Salmonella* challenge,^66^ as well as B cell–intrinsic TLR signalling; IgG production was dependent on T cells in this study, attributed to the spread of Gram‐negative commensal bacteria to systemic sites, including the spleen.

Although smaller in magnitude than IgG2b and IgG3 levels, a subset of microbiota is also targeted by a T cell–dependent IgG1 response (Figure 4), shown to be enriched for *Akkermansia muciniphila*,^124^ a widely‐studied commensal bacterium with potential therapeutic applications.^125^ *A muciniphila*‐specific TCR‐transgenic T cells demonstrated preferential T cell differentiation towards a Tfh phenotype within Peyer's patches when transferred into colonized mice, as well as a mixed Th phenotype within the gut lamina propria, including Th1, Th17 cells and Tregs. This T cell response was heavily microbiota‐dependent, with T cell fate differing substantially between altered Schaedler flora and SPF mice.^124^ Therefore, homeostatic mucosal IgG1 responses are linked to a subset of microbial species capable of inducing antigen‐specific adaptive immunity, unlike broadly polyreactive and T cell–independent IgA responses.^126^

Reductive experiments in which germ‐free mice were challenged with an auxotrophic non‐replicating *E coli* commensal strain enabled additional features of antigens that stimulate IgA vs IgG responses to be determined, beyond the nature of the bacterial strain, without the experimental interference of a pre‐existing host microbiome and antibody response.^127^, ^128^ In this model, recombinant dimeric monoclonal IgA antibodies derived from mucosal plasma cells exclusively bound plasma membrane bacterial antigens, and not cytoplasmic or ribosomal bacterial proteins.^129^ Intriguingly, another study using the same auxotrophic *E coli* strain confirmed that mucosal IgA is predominantly targeted to cell‐surface antigens, whilst IgG can target both intracellular and plasma membrane antigens.^128^ This suggests differing bacterial antigen processing may be required to generate IgA vs IgG responses. Therefore, although mucosal IgA and IgG have some overlapping antigen targets, there are clearly distinct mechanisms of action and signals required to mount mucosal IgA responses vs intestinal IgG responses.

### 5.2 Cytokine signals in IgA vs IgG responses

Several cytokines and soluble factors, including TGFβ, IL‐10, IL‐6 and retinoic acid, can promote IgA CSR in GALT. Of these, the most critical cytokine in Peyer's patches is TGFβ (Figure 4). Mice deficiency in TGFβRII exhibit abrogated IgA levels in Peyer's patches and elevated local and systemic IgG responses.^130^ Numerous cellular sources of TGFβ have been identified, such as T and B cells, FDCs and DCs, and it acts to induce expression of germ‐line α‐transcripts in B cells.^6^, ^131^ Recently, DCs within the subepithelial dome (SED) of Peyer's patches were found to play a critical role in IgA CSR through αvβ8‐mediated activation of TGFβ. Following activation, CCR6^+^ pre‐GC B cells migrate towards CCL20‐expressing follicle‐associated epithelial cells enabling interactions with DCs located in the SED that direct IgA CSR.^132^ Nitric oxide–producing DCs also support TD and TI IgA CSR through TGFβ receptor induction in B cells and DC‐intrinsic BAFF/APRIL expression, respectively. Beyond TGFβ, GALT DCs can also promote B cell gut tropism and IgA secretion through the production of RA in the absence of T cells.^133^ BAFF and APRIL are expressed within GALT and the lamina propria and can promote TI IgA CSR,^100^, ^134^, ^135^ although their primary role is suggested to be plasma cell maintenance.^4^, ^96^, ^100^, ^110^, ^136^ Their importance is highlighted by common variable immunodeficiency (CVID) and selective IgA deficiency (SIgAD) linked to mutations in *TNFRSF13B*, encoding TACI.^137^

It is notable that SIgAD is the most common primary immunodeficiency and remains largely asymptomatic in the majority of individuals. Compensation of other Ig subclasses, including IgG2, is required to prevent severe infections and complications.^138^ However, SIgAD patients are at increased risk of IgG‐associated disorders linked to a defective mucosal barrier, including coeliac disease, UC and autoimmunity.^137^, ^138^, ^139^ Therefore, intestinal penetrance of microbial and other environmental antigens seems to play an important role in mucosal and systemic IgG responses. However, little is known about the cytokine‐mediated signals that directly promote IgG class switching in the gut, although BAFF and APRIL can support IgG1 CSR in vitro.^134^, ^135^ Given the preferential induction of Peyer's patch‐resident Tfh cells by a subsets of the microbiota, IL‐4 and IL‐21 may play a role in mucosal IgG1 CSR in mice.^124^ However, very few commensal microbes have been identified that induce antigen‐specific IgA or IgG responses. Indeed, T cells appear dispensable for the majority of anticommensal IgA responses in health.^53^ Notably, circulating B cells and serum IgG/IgM levels are significantly reduced in humans with BAFF‐R deficiency, whilst these patients exhibit normal or high levels of IgA.^140^ However, further work is needed to identify the additional factors that promote homeostatic IgG responses in GALT.

## 6 IgG CLASS SWITCHING IN INFLAMMATORY BOWEL DISEASE

IBD is a chronic relapsing inflammatory disease of the gastrointestinal tract driven by an aberrant immune response against the microbiota. There are two major subtypes of IBD, Crohn's disease (CD) and ulcerative colitis (UC), which differ in their clinical presentations, genetic associations and determinant pathological immunity. CD may affect any part of the GI tract, most commonly the terminal ileum and colon, with inflammation occurring segmentally and transmural in nature.^141^ Genetic susceptibility to CD is associated with defects in microbial sensing and Th17 function (*NOD2*, *ATG16L1*, *LRRK2*, *IL23R* and *STAT3*).^142^ In contrast, UC targets the colon, with continuous superficial inflammation, and is genetically linked to alterations in barrier integrity (*HNF4A*) and the major histocompatibility complex region, Th17 function and *FCGR2A* polymorphisms.^143^

Beyond genetic susceptibility, microbial dysbiosis occurs in patients with IBD, with strong evidence indicating a role for the intestinal microbiota in triggering disease.^144^ In particular, lower bacterial diversity, a reduction in Bacteroides and Firmicutes bacteria, and an increase in Proteobacteria and Actinobacteria, is observed in CD, with similar changes reported in UC.^141^, ^143^, ^145^ Approximately, a third of CD patients have an increase in adherent‐invasive *Escherichia coli*,^146^ which have been shown to promote Th17 inflammation in vivo,^147^ whilst the presence of short‐chain fatty acid (SCFA)‐producing bacteria, such as *Bifidobacterium*, in CD patients is associated with quiescent disease and anti‐TNFα treatment response. This demonstrates a significant impact of microbial communities on the underlying immune response.^144^, ^145^

Although the pathogenic role of T cells and the IL‐23 pathway has been delineated, both clinically and in murine models of colitis,^142^, ^148^, ^149^, ^150^, ^151^ the role B cells and antibodies in IBD is much less well understood. The reported ineffectiveness of a grossly underpowered randomized controlled trial of rituximab (anti‐CD20 IgG) in the treatment of UC,^152^ which also represents a suboptimal strategy to deplete IgG‐producing plasma cells that do not express CD20,^153^ as well as case reports of de novo Crohn's disease following rituximab administration,^154^, ^155^ has led to a general conception that humoral immunity is unimportant in IBD. However, a combination of genetic,^142^, ^156^ single‐cell RNA sequencing^75^, ^157^, ^158^ and functional human and murine studies^72^, ^73^, ^74^, ^159^ support a pathogenic role for IgG in the pathogenesis of IBD. In particular, attention has centred on Fcγ receptors, given that a low‐affinity variant of the activating receptor FcγRIIA is linked to protection from UC and leads to attenuated myeloid cell responses to IgG.^72^, ^142^, ^156^ The genetic association of an IgG receptor and IBD is on the surface, counterintuitive, given the dominance of IgA in the intestine in health. However, we and others identified a marked increase in luminal, commensal‐binding IgG in UC,^8^, ^9^, ^10^, ^11^ suggesting a shift in the class‐switching signals encountered by intestinal B cells in IBD. Using two mouse models of intestinal inflammation, *Citrobacter rodentium,* a model of human attaching‐effacing *Escherichia coli* infection in humans, and dextran sodium sulphate (DSS) administration, we found a strong induction of IgG antibodies directed against the microbiota and enteropathogens.^66^, ^72^, ^74^ In these models, epithelial barrier breach induces local and systemic IgG that controls bacterial dissemination but may promote colitis through the activation of local FcγR‐expressing cells.^72^, ^73^, ^74^, ^75^ Specifically, FcyR‐expressing intestinal macrophages activation by commensal‐IgG immune complexes results in IL‐1β production, which in turn stimulates Th17 activation.^63^ Given the timescale of de novo IgG induction (typically beyond day 7) and the well‐known ability of DSS to induce disease in *Rag2*‐deficient mice, IgG is likely to predominantly contribute to chronic phases of inflammation in this model, although circulating anticommensal IgG is present in healthy mice that may be involved at disease onset.^53^, ^66^ Beyond *bone fide* infection models and chemically induced colitis, antimicrobial IgG is observed in a variety of spontaneous colitis models in immune‐replete mice, including *Il10* ^−/−^,^160^ C3H/HeJBir^161^ and *Nod2* ^−/−^ *Cybb* ^−/−^ mice,^162^ as well as mice strains that exhibit increased microbial penetrance, such as *Myd88* ^−/−^ *Ticam* ^−/−^ and *Nos2* ^−/−^ *Cybb* ^−/−^ double‐deficient mice.^163^ However, it remains to be determined whether IgG promotes intestinal inflammation across different murine models. Indeed, in *Nod2*/*Cybb* and *Nos2*/*Cybb* double‐deficient mice, the induction of microbiota‐targeting IgA and IgG antibodies is protective against microbial dissemination.^162^, ^163^

Whether the emergence of antimicrobial IgG is secondary to epithelial barrier dysfunction in IBD patients is not understood. It is noteworthy that anti‐microbial IgG is elevated in Crohn's disease patients several years prior to disease diagnosis,^164^ suggesting significant adaptive humoral immune dysregulation during very early disease. Moreover, IgG^+^ plasma cells are enriched in terminal ileum biopsies of newly diagnosed, early‐onset paediatric Crohn's disease patients.^75^ Therefore, it is likely that mucosal IgG responses exert differing effector functions across the timecourse of IBD, with variation in their detrimental contribution to inflammation.

Overall, the genetic signal, the prominent induction of intestinal IgG observed in IBD and subsequent functional experiments proving a causative role suggest that agents that could block IgG binding to activating FcγRs, or promote engagement or expression of the inhibitory FcyRIIB, may be useful therapeutic targets in IBD. In addition, the signals regulating the class switch to IgG observed in IBD may also represent a potential therapeutic target to prevent IgG generation and its subsequent inflammatory effector functions within the mucosa. Given their abundance in active disease, it is likely that T cells are involved in the IgG class‐switch response in IBD (Figure 4). Notably, clearance of *C rodentium* is critically dependent on B cells and IgG,^68^, ^165^, ^166^ with mice lacking IgG, but not IgM or secretory IgA, developing exacerbated intestinal pathology and succumb to systemic spread of infection. The IgG response specifically targets bacterial virulence factors and promotes pathogen eradication through activation of local myeloid cells,^165^ as well as IgG; CD4^+^ T cells are required for sterilizing immunity and *C rodentium*‐specific IgG responses are severely impaired in T cell–deficient mice.^166^ *Nod2*‐deficient animals also exhibit impaired IgG and IFNγ responses following *C rodentium* challenge.^167^ Given the role of IFNγ in promoting IgG CSR in vivo, this suggests a potential role for Th1 cells in intestinal IgG CSR.

T cells can also participate in shaping IgG glycosylation. As mentioned previously, IL‐23–mediated inflammation in mice has been shown to result in IgG desialylation that promotes inflammatory activity in models of rheumatoid arthritis.^107^ Indeed, IgG glycome profiling has identified decreased IgG sialylation in CD patients.^168^ Whether this directly impacts inflammatory IgG activity in IBD is not known but highlights a potential link between a major IBD‐associated risk pathway and local IgG induction. Furthermore, the impact of treatments, such as anti‐TNFα and anti‐IL‐23 monoclonal antibodies, on the frequency and inflammatory nature of IgG will be of great interest.

In addition to T cells, other immune cells and cytokines may impact the local IBD IgG response. In particular, BAFF expression is significantly increased in mucosal biopsies from IBD patients^169^ and represents a promising target in B cell–mediated diseases, such as lupus erythematosus and antibody‐mediated rejection.^170^ BAFF promotes B cell survival and augments B cell proliferation and Ig secretion following BCR engagement.^171^, ^172^, ^173^ BAFF also acts as a costimulatory factor in T cell activation,^169^ suggesting a potential role in targeting BAFF for dual T cell/B cell suppression. The nature of the IBD‐associated microbiota is likely to be critical in dictating B cell class switching. Certain bacterial species, such as adherent‐invasive *E coli*, which are prevalent in IBD, may influence T cell–dependent IgG responses in a manner analogous to invasive bacteria in mice. Furthermore, B cell responses may be directly or indirectly regulated by microbiota‐derived metabolites, such as SCFAs and aryl hydrocarbon receptor (AHR) ligands, which are known to be disrupted in IBD.^145^, ^174^, ^175^

The T‐box transcription factor family member T‐bet has traditionally been associated with initiating and directing effector Th1 responses and controlling IFN‐y secretion in viral infection, as well as in dictating Type 1 identity in ILC1s and a subset of mucosal ILC3s.^176^, ^177^ More recently, an atypical B cell subset expressing T‐bet and secreting IgG has received much attention due to their presence in both mice and humans in a variety of diseases, including autoimmune disorders such as multiple sclerosis and SLE, as well as infectious diseases including Hepatitis C, HIV and rhinovirus.^178^, ^179^, ^180^, ^181^, ^182^ B cell–specific T‐bet‐deficient mice demonstrated decreased class switching to IgG2a, and to a lesser extent, IgG2b and IgG3, in response to IFN‐y, as well as impaired IgG autoantibody production in a mouse model of lupus.^183^ Enforced expression of T‐bet ex‐vivo in T‐bet‐deficient B cells rescued germ‐line IgG2a transcription, suggesting a B cell–intrinsic role for T‐bet in IgG2a class switching.^105^ In mice immunized with the hapten NP‐KLH (nitrophenylacetyl‐keyhole limpet hemocyanin) following transfer of WT and *Tbx21* ^−/−^ splenic B cells, IgG2a^+^ memory B cell and plasma cells were reduced in the absence of T‐bet.^184^ A variety of overlapping factors have been proposed to promote T‐bet expression in B cells, mostly Type 1 cytokines including IL‐12, IL‐18, IFN‐y, IL‐27, as well as TLR7 and TLR9 agonists.^185^, ^186^, ^187^, ^188^ The presence of T‐bet‐expressing B cells is at least partially T cell contact‐ and antigen‐dependent, as they do not develop in the absence of MHC‐II or CD40 expression in B cells.^189^ Crucially, T‐bet^+^ B cells have been reported to be increased in frequency in the intestinal mucosa of CD, where their presence correlated with increased disease severity, suggestive of a proinflammatory contribution in CD.^190^ Moreover, these atypical T‐bet^+^ B cells were predominantly IgG^+^ and expressed higher amounts of IFN‐y than their IgA^+^ and IgM^+^ counterparts.^190^ IgA^+^ memory B cells do not rely on T‐bet for class switching and rather rely on the transcription factor RORα.^184^ This suggests that T‐bet, or the factors that drive its upregulation, may be a therapeutic target in IBD, as it is required for the generation of IgG^+^ B cells and CD4^+^ Th1 cells, both of which have pathogenic roles in IBD.

Recently, concurrent studies have identified the importance of anti‐fungal antibody responses in the gastrointestinal tract, expanding the role of humoral intestinal immunity into the eukaryotic kingdom. The presence of specific serum IgG against fungal species has been well‐documented in Crohn's disease patients, specifically anti‐*Saccharomyces cerevisiae* antibodies (ASCAs).^191^, ^192^ However, mucosal Ig responses to fungal species are distinct from those in the serum,^193^ predominantly targeting *Candida* species, particularly the pathobiont *C albicans*, which may be pathogenic, causing fatal extraintestinal diseases, including meningoencephalitis in some contexts.^194^ Intriguingly, parallel studies investigating adaptive immunity‐dependent mucosal responses in mice against *C albicans* (mostly IgA with some IgG1) have shown they are preferentially targeted against antigens expressed only on the tissue‐invasive hyphal morphotype of the fungus and not the less virulent circular yeast form.^195^, ^196^ These hyphae‐expressing *C albicans* are associated with worse colitis and extraintestinal diseases. In a cohort of 12 patients with Crohn's disease and 9 healthy controls, the targeting of these hyphal‐associated virulence factors by intestinal antibodies was decreased in CD patients, leading to increased hyphal forms of fungi.^195^ Thus, by selectively targeting for pathogenic forms of the same fungus over commensal forms, the intestinal humoral immune response can promote homeostasis and ward off distinct morphotypes that are pathogenic if they gain access to the systemic system.^197^ Similar disparate antibody responses to distinct genetic phase variations of a single bacterium species have also been reported.^129^

Although, at baseline, intestinal fungi are bound by only low amounts of mucosal IgG, this can be increased rapidly by adding e*x vivo* of serum IgG in both humans and mice.^193^ This IgG is antigen‐specific, dependent on T cell presence for its generation and significantly lowered in germ‐free mice. IgG2b and IgG3 constitute the majority of serum IgG capable of binding to commensal gut fungi in mice. This IgG‐bound mycobiota targets predominantly *Candida albicans*, with no other strains, including *S cerevisiae,* capable of generating serum IgG responses in germ‐free mice. Intriguingly, the site of B cell expansion and IgG isotype switching was found to be not the Peyer's patches or the mesenteric lymph nodes, but the spleen, which accumulated more Fas^+^GL‐7^+^ germinal centre B cells and IgG^+^ B cells following *C albicans* colonization.^193^ This suggest that controlled movement of gut‐resident antigens and/or B cells to extraintestinal lymphoid tissues can educate extraintestinal humoral immunity and induce systemic mucosal‐educated IgG antibodies with targeted specificity for gut fungi, in a similar manner has been reported for IgA gut‐educated antibodies.^198^, ^199^, ^200^ Depletion of intestinal CXC3R1^+^ MNPs in mice disrupts systemic anti‐*C albicans* IgG and polymorphisms in the coding region of *CXC3R1* in humans have been associated with increased antifungal systemic IgG in CD patients.^201^ Whether these systemic IgG antibodies can subsequently gain access to the intestinal mucosa during periods of inflammation is unknown.

## 7 MUCOSAL PLASMA CELL NICHES

Beyond IgG CSR, the gut‐trophic factors and distinct mucosal niches occupied by IgG^+^ and IgA^+^ plasma cells, may represent another therapeutic target in intestinal disease. For example, IgG^+^ plasma cells are enriched in inflamed intestinal tissue across a range of disorders (HIV infection, chronic granulomatous disease and CD) and exhibit unique chemokine receptor expression, with reduced CCR10 and increased CXCR4 expression relative to IgA plasma cells.^202^, ^203^

Broadly speaking, the chemokine CCL25 is required for the recruitment of IgA^+^ plasma cells into the lamina propria of the small intestine, whilst CCL28 is more important for localizing plasma cells in the large intestine,^204^, ^205^, ^206^ with eosinophils implicated as a niche component for IgA^+^ plasma cells,^136^ and this differs from the niche requirements of IgG plasma cells. For example, Salmonella‐specific IgA plasmablasts generated by oral vaccination in humans display robust migration towards the mucosal‐associated cytokine CCL28 in exvivo transwell assays, in contrast to Salmonella‐specific IgG plasmablasts, which showed little CCL28‐dependent chemotaxis.^207^ Consistent with this, CCL28 is abundantly expressed in both intestinal and extraintestinal mucosal tissues, and its receptor CCR10 is highly expressed by IgA^+^ plasmblasts.^204^, ^208^ Intestinal IgG^+^ B cells preferentially migrated towards CXCL11^209^ expressed by monocytes in response to microbial stimulation and overexpressed in inflamed tissue in IBD patients.^210^, ^211^ Collectively, these studies suggest that distinct chemokine cues orchestrate isotype‐specific B cell and plasmablast movement and residency to specific intestinal niches in health and disease.

In the human colon, IgG^+^ plasma cells are enriched in the distal sigmoid colon,^33^ which suggests that niches for these cells could be influenced by bacterial diversity or the local Th1:Th17 ratio. In addition, the distribution of commensal‐sensing luminal‐facing epithelial cell receptors shows non‐uniform distribution along the intestine, highlighting areas where host‐microbiota immunological interfaces are more likely to occur.^212^ Local IgG‐commensal immune complexes may also stimulate mononuclear phagocytes to provide IgG plasma cell niche factors, as myeloid cells have been well‐described as plasma cell niche participants in the bone marrow, via surface CD80/86 expression and cytokines such as IL‐6.^213^ Single‐cell transcriptomic studies have identified cellular modules associated with IgG plasma niches in CD patients refractory to anti‐TNF therapy.^158^ Further delineation of the specific factors, which initiate and maintain intestinal IgG^+^ plasma cell niches, could enable therapeutic targeting to disrupt the production of pathogenic IgG^+^ in IBD whilst sparing beneficial homeostatic IgA‐producing cells.

## 8 CONCLUSION

Despite the renewed interest in recent years in mucosal IgG, much work is needed to elucidate the mechanisms that regulate the induction of IgG responses in health, enteric infection and chronic inflammatory disease within the gastrointestinal tract. Whilst IgA is clearly the dominant isotype during homeostasis, low‐level IgG production occurs continuously against certain constituents of the microbiome,^53^, ^124^ providing systemic protection against infection,^66^ and is significantly induced during infection and colitis.^33^, ^68^, ^72^ Continued dissection of the molecular mechanisms regulating IgG class switching in the GALT and mucosa will help identify potential targets to manipulate the course of intestinal infection or chronic inflammation.

## CONFLICT OF INTEREST

The authors declare no competing interests.

## Data Availability

Data sharing is not applicable to this article as no new data were created or analyzed in this study.
